# ZFP36 Regulates Vascular Smooth Muscle Contraction and Maintains Blood Pressure

**DOI:** 10.1002/advs.202408811

**Published:** 2024-11-26

**Authors:** Xiuru Cui, Yawei Wang, Hanlin Lu, Lei Wang, Xianwei Xie, Shenghao Zhang, Pavel Kovarik, Shuijie Li, Shanshan Liu, Qunye Zhang, Jianmin Yang, Cheng Zhang, Jinwei Tian, Yan Liu, Wencheng Zhang

**Affiliations:** ^1^ State Key Laboratory for Innovation and Transformation of Luobing Theory Key Laboratory of Cardiovascular Remodeling and Function Research Chinese Ministry of Education Chinese National Health Commission and Chinese Academy of Medical Sciences Department of Cardiology Qilu Hospital of Shandong University Jinan 250012 China; ^2^ Department of Cardiology Second Affiliated Hospital of Harbin Medical University Heilongjiang Provincial Key Laboratory of Panvascular Disease The Key Laboratory of Myocardial Ischemia Ministry of Education Harbin 150086 China; ^3^ Max Perutz Labs University of Vienna Vienna Biocenter (VBC), Dr. Bohr‐Gasse 9 Vienna A‐1030 Austria; ^4^ Department of Biopharmaceutical Sciences College of Pharmacy Harbin Medical University Harbin 150081 China; ^5^ State Key Laboratory of Transvascular Implantation Devices Heart Regeneration and Repair Key Laboratory of Zhejiang Province Department of Cardiology The Second Affiliated Hospital School of Medicine Zhejiang University Hangzhou 310009 China

**Keywords:** blood pressure, hypertension, smooth muscle contraction, ZFP36

## Abstract

Hypertension remains a major risk factor for cardiovascular diseases, but the underlying mechanisms are not well understood. Zinc finger protein 36 (ZFP36) is an RNA‐binding protein that regulates mRNA stability by binding to adenylate‐uridylate‐rich elements in the mRNA 3′‐untranslated region. This study reveals that ZFP36 expression is highly elevated in the arteries of hypertensive patients and rodents. In cultured vascular smooth muscle cell (VSMC), angiotensin II (AngII) activates poly (ADP‐ribose) polymerases1 (PARP1) to stimulate *Zfp36* expression at the transcriptional level. VSMC‐specific ZFP36 deletion reduces vessel contractility and blood pressure levels in mice. Mechanistically, ZFP36 regulates G protein‐coupled receptors (GPCRs)‐mediated increases in intracellular calcium levels through impairing the mRNA stability of regulator of G protein signaling 2 (RGS2). Moreover, the VSMC‐specific ZFP36 deficiency attenuates AngII‐induced hypertension and vascular remodeling in mice. AAV‐mediated ZFP36 knockdown ameliorates spontaneous hypertension in rats. These findings elucidate that ZFP36 plays an important role in the regulation of smooth muscle contraction and blood pressure through modulating RGS2 expression. ZFP36 inhibition may represent a new therapeutic strategy for the treatment of hypertension.

## Graphical abstract

This research investigates the role and mechanism of the RNA‐binding protein ZFP36 in regulating blood pressure and smooth muscle contraction. VSMC‐specific ZFP36 deletion reduces vessel contractility and blood pressure in mice. Mechanistically, ZFP36 regulates intracellular calcium levels through impairing the mRNA stability of RGS2. ZFP36 inhibition represents a new therapeutic strategy for treating hypertension.

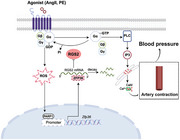

## 1 Introduction

Hypertension is a common chronic disease impacting 1.4 billion people worldwide, accounts for 10.4 million deaths each year.^[^ ^1^ ^]^ Hypertension causes severe damage to many organs, including the brain, heart, and kidneys, and it is the main risk factor for stroke and coronary artery disease. Thus, the mechanisms underlying hypertension should be urgently explored, and novel therapeutic targets and strategies should be developed. Blood pressure is affected by blood volume, cardiac output, and peripheral resistance, which depend on the balance between the contraction and relaxation of vascular smooth muscle cells (VSMCs). Abnormalities in the VSMC contractile state can cause disorders of blood pressure, including hypertension.^[^ ^2^ ^]^ However, the detailed mechanism of vascular contractility has not been well defined.

An increase in intracellular calcium (Ca^2+^) is a key step in initiating VSMC contraction, and a variety of Ca^2+^‐permeable channels orchestrate the dynamic and precise control of intracellular Ca^2+^ concentration. Spatial and temporal changes in intracellular Ca^2+^ are regulated by Ca^2+^ influx through voltage‐dependent and independent plasmalemmal Ca^2+^‐permeable channels, as well as Ca^2+^ release from intracellular stores.^[^ ^3^ ^]^ L‐type Ca^2+^ channels have long been considered the primary route of Ca^2+^ entry into VSMCs. In addition to these channels, T‐type Ca^2+^ channels are emerging as important contributors to myogenic tone.^[^ ^4^ ^]^ G protein‐coupled receptors (GPCRs) can be stimulated and activated by agonists, leading to inositol 1,4,5‐trisphosphate formation and subsequent Ca^2+^ release from the sarcoplasmic reticulum. The increased cytosolic Ca^2+^ binds with calmodulin, which activates myosin light chain kinase, thereby resulting in the phosphorylation of the regulatory light chain of smooth muscle myosin and subsequent contraction.^[^ ^5^ ^]^ Regulator of G protein signaling (RGS) proteins play a crucial role in G protein signaling by acting as GTPase‐activating proteins, accelerating the rate that Gα subunits hydrolyse GTP and consequently deactivating signal transduction.^[^ ^6^ ^]^ Some members of the RGS protein family show high expression in the cardiovascular system and are involved in cardiovascular function, for example RGS2, which is a key regulator of vascular contractility and blood pressure.^[^ ^7^, ^8^ ^]^ However, the mechanisms by which these proteins regulate RGS expression have not been well studied.

The post‐transcriptional regulation of genes by RNA‐binding proteins, particularly mRNA stability, enables rapid changes in mRNA levels. Dysregulated mRNA stability could contribute to the development of diseases.^[^ ^9^ ^]^ In our previous study, we found that downregulated RGS proteins in RNA‐binding protein HuR‐deficient mice resulted in hypertension,^[^ ^10^ ^]^ which suggests the important role of RNA‐binding proteins. Zinc finger protein 36 (ZFP36), encoded by the *Zfp36* gene, is another RNA‐binding protein containing a tandem Cys‐Cys‐Cys‐His zinc finger domain. The function of ZFP36 is to decay mRNA by binding to adenylate/uridylate‐rich‐elements in the 3′ untranslated regions of its target mRNAs.^[^ ^11^ ^]^ Although ZFP36 plays crucial roles in macrophages,^[^ ^12^ ^]^ cardiomyocytes,^[^ ^13^ ^]^ tumour cells,^[^ ^14^, ^15^ ^]^ etc., its function in VSMCs is unclear.

Therefore, in this study, we generate VSMC‐specific ZFP36‐knockout (*Zfp36^SMKO^*) mice to investigate the role of ZFP36 in the regulation of VSMC contraction and blood pressure. Our results show that *Zfp36^SMKO^* mice display reduced blood pressure and decreased vasoconstrictor responses to vasoconstrictor substances.

## 2 Results

### 2.1 ZFP36 is Increased in the Hypertensive Arteries

**Figure 1:**
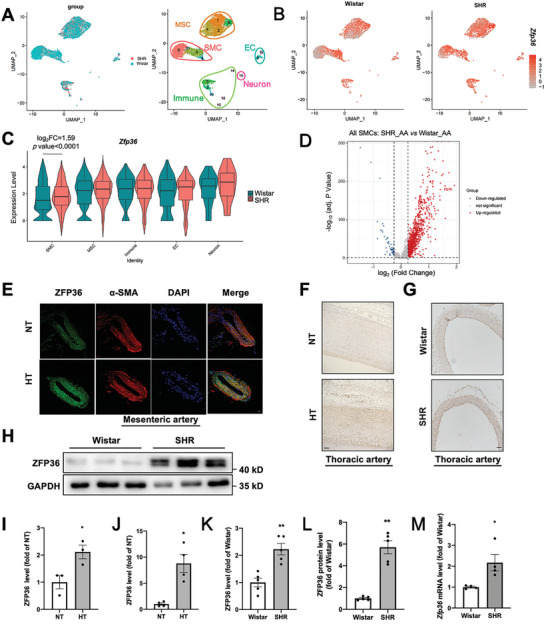
ZFP36 levels were significantly elevated in patients and animals with hypertension. A) ScRNA sequencing datasets and cell subpopulation annotation after integration of SHR and Wistar groups of rat aortic tissue. B) Feature plot of *Zfp36* expression in aortic tissue of Wistar and SHR rats. C) Violin plot of *Zfp36* expression in different cell types between SHR and Wistar groups. D) Volcano plot of differentially expressed genes in all smooth muscle cells (SMCs; average log_2_ fold change >0.25, adjusted *P* < 0.05). E) Immunofluorescent staining of ZFP36 protein in the mesenteric artery of patients with hypertension and normotensive controls (*n =* 3). α‐SMA indicates α smooth muscle actin, and DAPI indicates 4,6'‐diamidino‐2‐phenylindole. Scale bar: 100 µm. F) Immunohistochemical staining of ZFP36 in the thoracic artery of patients with hypertension and normotensive controls. Scale bar: 100 µm (*n =* 4). G) Immunohistochemical staining of ZFP36 in the thoracic artery of spontaneously hypertensive (SHR) and Wistar rats. Scale bar: 100 µm (*n =* 5). H) Western blot analysis of ZFP36 expression in aortas of Wistar and SHR rats (*n =* 5). I–L) Quantitative analysis of ZFP36 levels in (E–H), ^*^ *P* < 0.05, ^**^ *P* < 0.01 *vs* NT/Wistar. M) RT‐PCR analysis of *Zfp36* mRNA levels in aortas of Wistar and SHR rats (*n =* 4). ^*^ *P* < 0.05 *vs* Wistar. Data were expressed as the mean ± SEM. Two‐tailed Student's unpaired *t*‐test was used for analysis in (I–M).

To explore the role of ZFP36 in hypertension, we first re‐analyzed single‐cell sequencing data of the aorta of SHR and Wistar rats from the GSE149777 dataset. The unbiased clusters were annotated to major vascular cell types according to known marker genes: smooth muscle cells (SMCs; Myh11 and Cnn1; clusters 0, 3, 8 and 11), endothelial cells (ECs; Cdh5 and Vwf; clusters 9 and 12), mesenchymal stromal cells (MSCs; Gpx3, and Dcn; clusters 1, 2, 4, and 6), immune cells (Ptprc; clusters 5, 7, 10, 13, 14 and 16), and neurons (Plp1, clusters 15) (**Figure** **1**A). The feature, violin, and volcano plots indicate that *Zfp36* expression was increased in the SMCs clusters of the arteries from SHR rats compared to Wistar rats (Figure 1B–D).

To further confirm the role of ZFP36 in the pathogenesis of hypertension, we analyzed its expression in the arteries of patients and rats with hypertension. Immunofluorescence (Figure 1E,I) and immunohistochemistry staining (Figure 1F,J) results revealed that ZFP36 levels were significantly higher in the mesenteric arteries and aortas of patients with hypertension than in those with normotension. In addition, immunohistochemical staining (Figure 1G,K) and western blotting (Figure 1H,L) results confirmed that ZFP36 protein levels were higher in the aortas of SHR rats than in those of Wistar rats, which is consistent with the increased mRNA levels (Figure 1M). These results suggest that high ZFP36 expression may contribute to the pathogenesis of hypertension.

### 2.2 AngII Induces ZFP36 Expression in VSMCs

**Figure 2:**
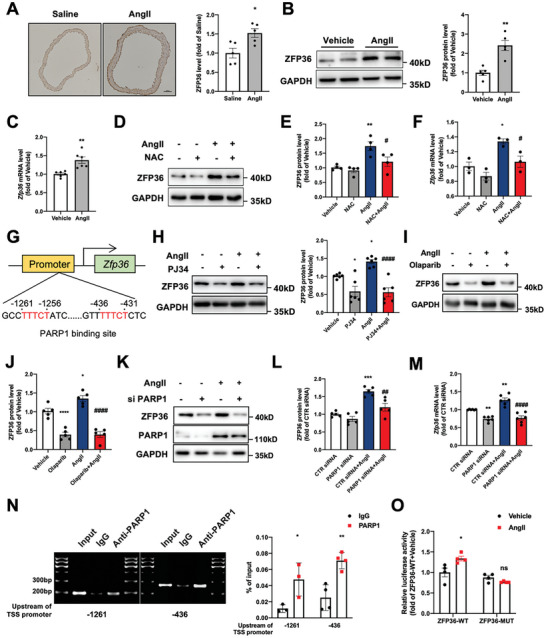
Angiotensin II upregulated ZFP36 expression in vascular smooth muscle cells (VSMCs). A, Mice were treated with saline or AngII for four weeks followed by immunohistochemical staining of ZFP36 protein in the thoracic artery; representative images are shown on the left, and quantitative data are shown on the right (*n* = 5). Scale bar: 100 µm. *P < 0.05 *vs* Saline. B‐C, VSMCs were treated with AngII (0.1 µmol L^−1^) for 24h followed by western blot analysis (*n* = 5) and quantitative RT‐PCR analysis (*n* = 6) of Zfp36 expression. **P < 0.01 *vs* vehicle. D‐F, VSMCs were pretreated with N‐acetylcysteine (1 mmol L^−1^) for 30 min and then stimulated with AngII followed by western blot (D and E, *n* = 4) and RT‐PCR (F, *n* = 3) analysis of ZFP36 expression. *P < 0.05, **P < 0.01 *vs* vehicle, ^#^ *P* < 0.05 *vs* AngII. G, Predicted PARP1 binding sites in the mouse *Zfp36* promoter. H, VSMCs were treated with PJ34 (10 µmol L^−1^) and AngII (0.1 µmol L^−1^) for 24h, followed by western blot analysis of ZFP36 expression (*n* = 6). *P < 0.05 *vs* vehicle, ^###^ *P* < 0.001 *vs* AngII. I‐J, VSMCs were treated with olaparib (10 µmol L^−1^) and AngII (0.1 µmol L^−1^) for 24h, western blot analysis of ZFP36 expression (*n* = 5). *P < 0.05, ****P < 0.0001 *vs* vehicle, ^####^ *P* < 0.0001 *vs* AngII. K‐M, VSMCs were transfected with control siRNA or PARP1 siRNA, and then treated with AngII (0.1 µmol L^−1^) for 24h, western blot analysis (K and L, *n* = 5) and quantitative RT‐PCR analysis (M, *n* = 6) of ZFP36 expression was performed. **P < 0.01, ***P < 0.001 *vs* Vehicle, ^##^ *P* < 0.01, ^####^ *P* < 0.0001 *vs* AngII. N, Chromatin immunoprecipitation (ChIP) assays were performed by using anti‐PARP1 or rabbit IgG as an isotype control in VSMCs. Enrichment of PARP1 binding to the predicted PARP1 binding sites in the promoter regions of Zfp36 was quantified by PCR with primers, *n* = 3 or 4, *P < 0.05, **P < 0.01 *vs* IgG. O, VSMCs were transfected with wild‐type (WT) and mutant constructs of *Zfp36* promoter, together with PRL‐TK, after treatment with AngII for 24h, and the luciferase activity was measured (*n* = 4), *P < 0.05 *vs* ZFP36‐WT + Vehicle. Data were expressed as the mean ± SEM. Two‐tailed Student's unpaired t‐test was used for analysis in A‐C, One‐way ANOVA followed by Tukey's post‐test analysis was used for E, F, H‐J, L‐O.

Given the critical role of AngII and homocysteine (Hcy) in hypertension and vascular diseases, whether they regulated ZFP36 expression was determined. We first observed that ZFP36 expression was increased in the aorta of mice treated with AngII for four weeks (**Figure** **2**A). In vitro, we isolated and cultured the mouse primary VSMCs, which was validated by immunofluorescence staining with α‐SMA (Figure S1A, Supporting Information). AngII upregulated ZFP36 protein and mRNA levels in primary VSMCs (Figure 2B,C). Moreover, Hcy also could elevate ZFP36 expression in VSMCs (Figure S1B,C, Supporting Information). Oxidative stress was the primary factor leading to vascular damage under AngII stimulation,^[^ ^16^ ^]^ and the antioxidant effects of N‐acetylcysteine (NAC) significantly prevented AngII‐induced ROS levels (Figure S1D, Supporting Information) and ZFP36 upregulation (Figure 2D–F). To investigate the mechanism underlying the AngII‐induced upregulation of ZFP36, we used the Transcription Factor Database (http://jaspar.genereg.net) to predict two putative binding sites specific for PARP1 in the *Zfp36* promoter (−1,261 bp and −436 bp upstream of the transcription start sites) (Figure 2G). Next, we examined whether AngII‐induced ZFP36 transcription occurred via PARP1. Inhibition of PARP1 using its selective inhibitors PJ34 and olaparib impaired the AngII‐induced increase in ZFP36 (Figure 2H–J). PARP1 knockdown by siRNA also blocked the AngII‐induced upregulation of ZFP36 in VSMCs (Figure 2K–M). To further confirm the involvement of PARP1 in ZFP36 regulation, ChIP revealed that PARP1 could directly bind to the *Zfp36* promoter (Figure 2N). Moreover, DNA pull down assay further validated that PARP1 could bind to the *Zfp36* promoter in vitro (Figure S1E). In addition, AngII increased *Zfp36* promoter luciferase activity; however, this activation was abolished in the PARP1 binding sites of the mutant promoter (Figure 2O). Thus, AngII upregulated ZFP36 expression through PARP1 in VSMCs.

### 2.3 VSMC‐Specific Deletion of ZFP36 Reduces Blood Pressure in Mice

**Figure 3:**
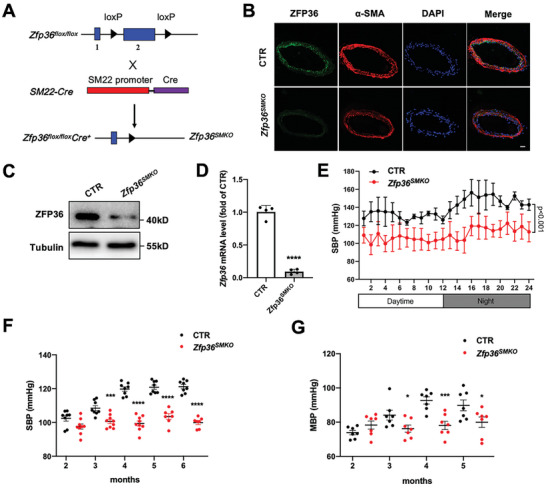
VSMC‐specific knockout of ZFP36 reduced blood pressure. A) Generation of VSMC‐specific ZFP36‐knockout (*Zfp36^SMKO^*) mice. B) Immunofluorescent staining of ZFP36 protein in mesenteric artery of control (CTR) and *Zfp36^SMKO^* mice. α‐SMA indicates α smooth muscle actin, and DAPI indicates 4,6'‐diamidino‐2‐phenylindole. Scale bar: 20 µm. C) Western blot analysis of ZFP36 in mesenteric artery of CTR and *Zfp36^SMKO^* mice. D) RT‐PCR analysis of *Zfp36* mRNA levels in aortas of CTR and *Zfp36^SMKO^* mice (*n =* 4), ^****^ *P* < 0.0001 *vs* CTR. E) Systolic blood pressure levels in four‐month‐old CTR and *Zfp36^SMKO^* mice were monitored by 24‐h telemetry (*n =* 4). F) Measurement of systolic blood pressure (SBP) in male CTR and *Zfp36^SMKO^* mice at different ages (*n =* 7–9). G) Mean blood pressure (MBP) in male CTR and *Zfp36^SMKO^* mice at different ages (*n =* 7). ^*^ *P* < 0.05, ^***^ *P* < 0.001, ^****^ *P* < 0.0001 *vs* CTR. Data were expressed as the mean ± SEM. Two‐tailed Student's unpaired t‐test was used for D, F, and G, repeated measures ANOVA was used for (E).

To explore the role of ZFP36 in smooth muscles, we generated VSMC‐specific *Zfp36*‐knockout mice. To specifically delete the *Zfp36* gene in VSMCs, *Zfp36*‐floxed (*Zfp36^flox/flox^*) mice were crossbred with *SM22‐Cre* transgenic mice to obtain *SM22‐Cre*/*Zfp36^flox/+^* mice, which were further inter‐crossed to generate *Zfp36^flox/flox^*/*Cre^+^* mice (referred to as *Zfp36^SMKO^*; **Figure** **3**A), with littermate *Zfp36^flox/flox^*/*Cre^−^* mice as the control group. Western blotting and immunofluorescence staining confirmed ZFP36 deficiency in the mesenteric arteries of *Zfp36^SMKO^* mice (Figure 3B,C). *Zfp36* mRNA levels were reduced in the aortas from *Zfp36^SMKO^* mice (Figure 3D). There was no significant difference in media layer thickness of thoracic arteries between the control and *Zfp36^SMKO^* mice (Figure S2A, Supporting Information). To characterize the phenotype of *Zfp36^SMKO^* mice, blood pressure was measured using radiotelemetry, which showed that *Zfp36^SMKO^* mice had a lower systolic blood pressure (Figure 3E). The results of the tail‐cuff test also revealed that *Zfp36^SMKO^* mice had lower systolic and mean blood pressures at three months of age (Figure 3F,G). However, there were no differences in diastolic blood pressure and heart rate between the male control and *Zfp36^SMKO^* mice (Figure S2B,C, Supporting Information). Meanwhile, female *Zfp36^SMKO^* mice also had lower systolic blood pressure and mean blood pressure than control, but no significant difference in diastolic blood pressure (Figure S2D–F, Supporting Information). These results suggest that the deletion of ZFP36 in smooth muscle reduces blood pressure in mice.

### 2.4 Deletion of ZFP36 Reduces the Contractile Responses of Mesenteric Arteries

**Figure 4:**
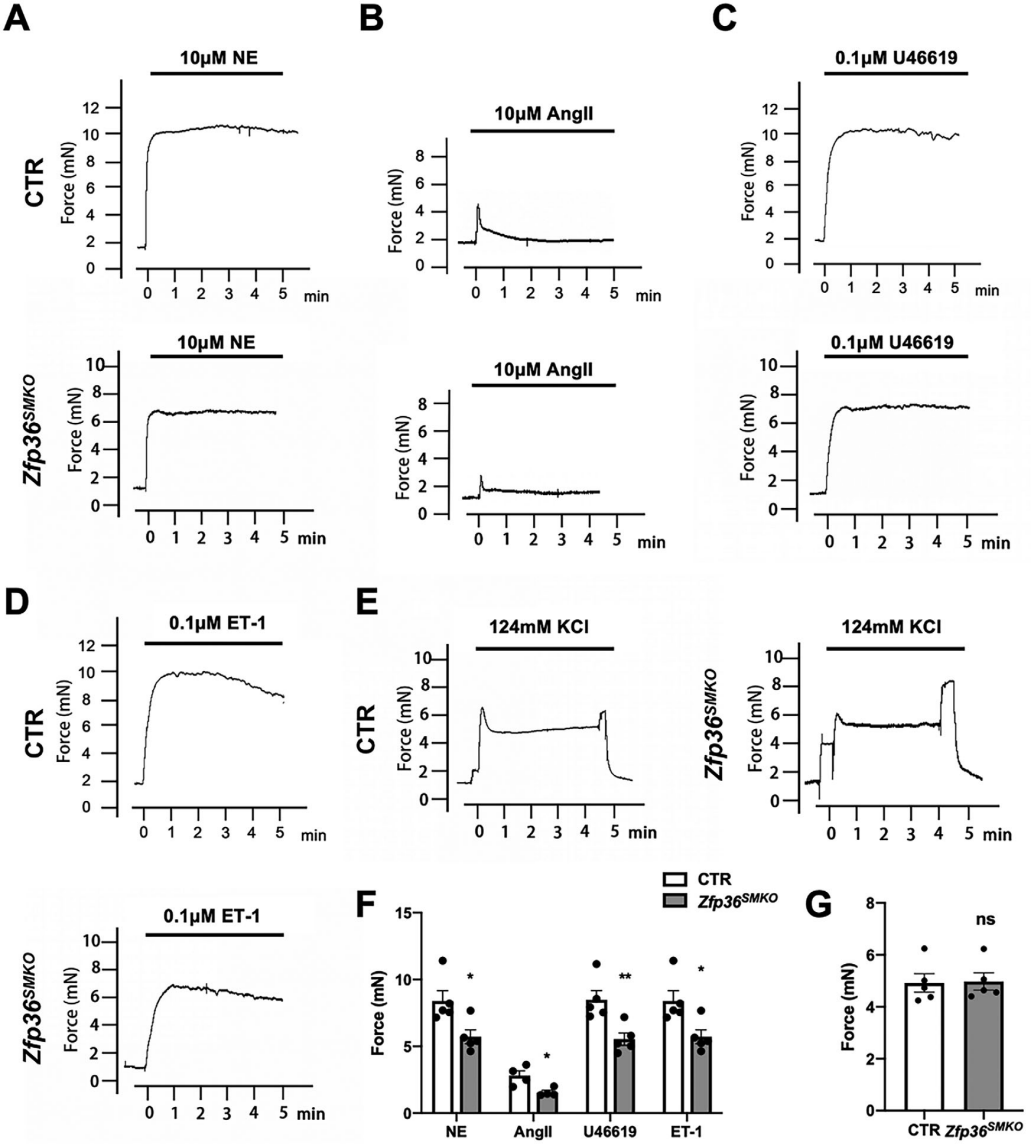
Deletion of ZFP36 decreased VSMC contractile responses. A–E) Mesenteric arteries from CTR and *Zfp36^SMKO^* mice were mounted in a wire myograph, and contractile response curves were generated over 5 min for (A) NE (norepinephrine, 10 µmol L^−1^), B) AngII (10 µmol L^−1^), (C) U46619 (0.1 µmol L^−1^), D) ET‐1 (endothelin‐1, 0.1 µmol L^−1^) and E) KCl (124 mmol L^−1^). F,G) Quantified peak force responses with vasoconstrictors (*n* = 4 or 5). ^*^ *P* < 0.05, ^**^ *P* < 0.01 *vs* CTR. Data were expressed as the mean ± SEM. Two‐tailed Student's unpaired *t*‐test was used for F, G.

Blood pressure is closely related to peripheral resistance, which is mainly caused by the small arteries. To determine the importance of ZFP36 for the contractile function of small arteries, secondary branches of the mesenteric arteries were isolated and there was no significant difference in the mesenteric artery diameter between control and *Zfp36^SMKO^* mice (10.10 ± 0.31 versus 10.03 ± 0.33 µm). Then, mesenteric arteries were mounted in wire myographs to generate response curves for the vasoactive agonists NE, AngII, U46619, and ET‐1. The results showed that contraction of the mesenteric arteries from *Zfp36^SMKO^* mice in response to all four vasoactive agonists was weaker than that of the control arteries (**Figure** **4**A–D,F). Intriguingly, we observed no significant difference in the response to KCl‐induced depolarisation between control and *Zfp36^SMKO^* arteries (Figure 4E,G). Therefore, contractile responses elicited by GPCRs appeared to be more sensitive to ZFP36 deficiency than those elicited by membrane depolarisation. The decreased contractile response to vasoactive agonists may contribute to reduced blood pressure in *Zfp36^SMKO^* mice.

### 2.5 ZFP36 Reduces RGS2 Expression

**Figure 5:**
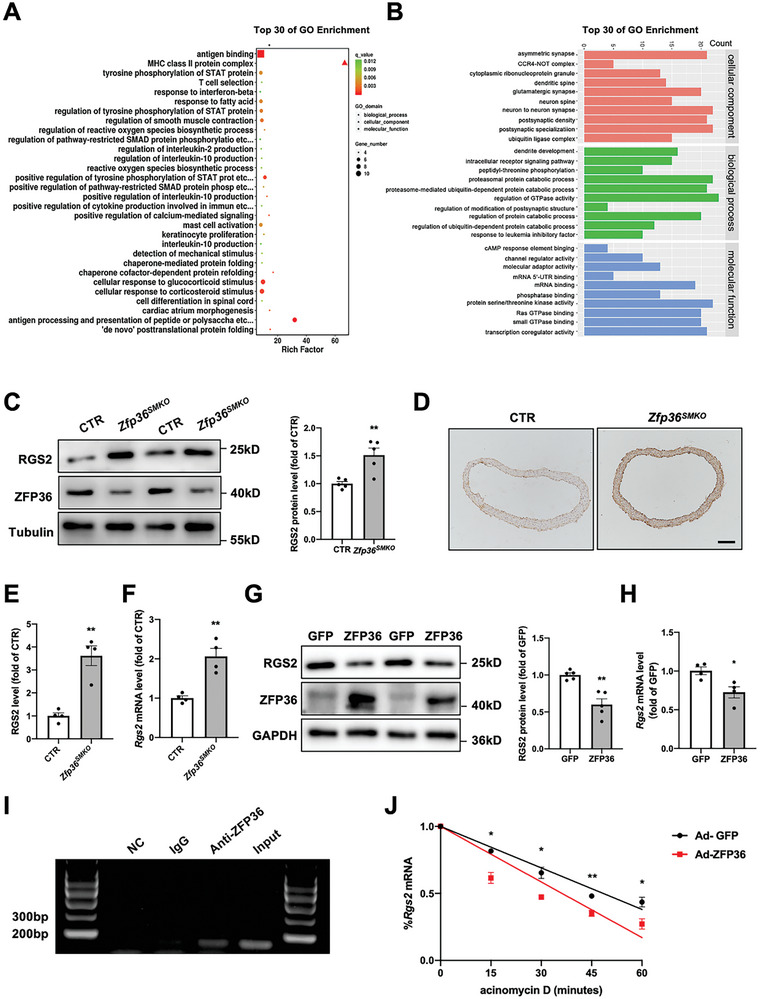
ZFP36 reduced regulator of G protein signaling 2 (RGS2) expression. A) Gene Ontology analysis of dysregulated genes in CTR and *Zfp36^SMKO^* mice by RNA sequencing. B) Gene Ontology analysis of different genes by RIP sequencing. C) Western blot analysis of RGS2 in aortas of CTR and *Zfp36^SMKO^* mice (*n =* 5). ^**^ *P* < 0.01 *vs* CTR. D) Immunohistochemical staining of ZFP36 protein in thoracic artery. Scale bar: 100 µm. E) Quantitative analysis of (D) (*n =* 4). ^**^ *P* < 0.01 *vs* CTR. F) RT‐PCR analysis of *Rgs2* mRNA levels in aortas of CTR and *Zfp36^SMKO^* mice (*n =* 4). ^**^ *P* < 0.01 *vs* CTR. G,H) VSMCs were infected with adenovirus‐expressing green fluorescent protein (GFP) or ZFP36 for 48 h followed by western blot analysis (G, *n =* 5) and RT‐PCR analysis (H, *n =* 4) of RGS2. ^*^ *P* < 0.05, ^**^ *P* < 0.01 *vs* GFP. I) RNA immunoprecipitation with anti‐ZFP36 antibody or control IgG antibody was performed followed by PCR analysis. J) VSMCs were infected with adenovirus‐expressing GFP or ZFP36, then treated with actinomycin D (5 µg mL^−1^). RT‐PCR analysis of *Rgs2* mRNA levels was performed. ^*^ *P* < 0.05, ^**^ *P* < 0.01 *vs* Ad‐GFP. Data were expressed as the mean ± SEM. Two‐tailed Student's unpaired *t*‐test was used for (C, E‐H, and J).

To explore the underlying mechanism by which VSMC ZFP36 regulates arterial contractility, RNA sequencing was performed to determine the differences in gene expression between control and *Zfp36^SMKO^* aortas. The top 30 pathways determined by Gene Ontology enrichment analysis revealed that ZFP36 knockout was closely related to VSMC contraction (**Figure** **5**A). RIP sequencing with an anti‐ZFP36 or IgG antibody was also performed; according to Gene Ontology enrichment analysis, the mRNAs to which ZFP36 binds are involved in regulating GTPase activity (Figure 5B). RGS2 is a well‐known GTPase‐activating protein closely related to vasoconstriction. We found that RGS2 protein levels were significantly increased in the aortas of *Zfp36^SMKO^* mice (Figure 5C), which is consistent with the increased mRNA levels (Figure 5F). Furthermore, immunohistochemical staining confirmed that ZFP36 deletion increased RGS2 expression (Figure 5D,E). Moreover, ZFP36 overexpression reduced the protein and mRNA levels of RGS2 in VSMCs (Figure 5G,H). To further confirm that ZFP36 bound to *Rgs2* mRNA, RIP‐PCR was performed (Figure 5I). Next, we examined the effect of ZFP36 on the stability of *Rgs2* mRNA, the result showed that the half‐life of *Rgs2* mRNA was markedly decreased upon ZFP36 overexpression in VSMCs (Figure 5J). Overall, these results suggested that ZFP36 downregulates RGS2 expression by binding to its mRNA and decreasing its stability.

### 2.6 ZFP36 Regulates GPCR‐Mediated Intracellular Ca^2+^ Increase Through RGS2

**Figure 6:**
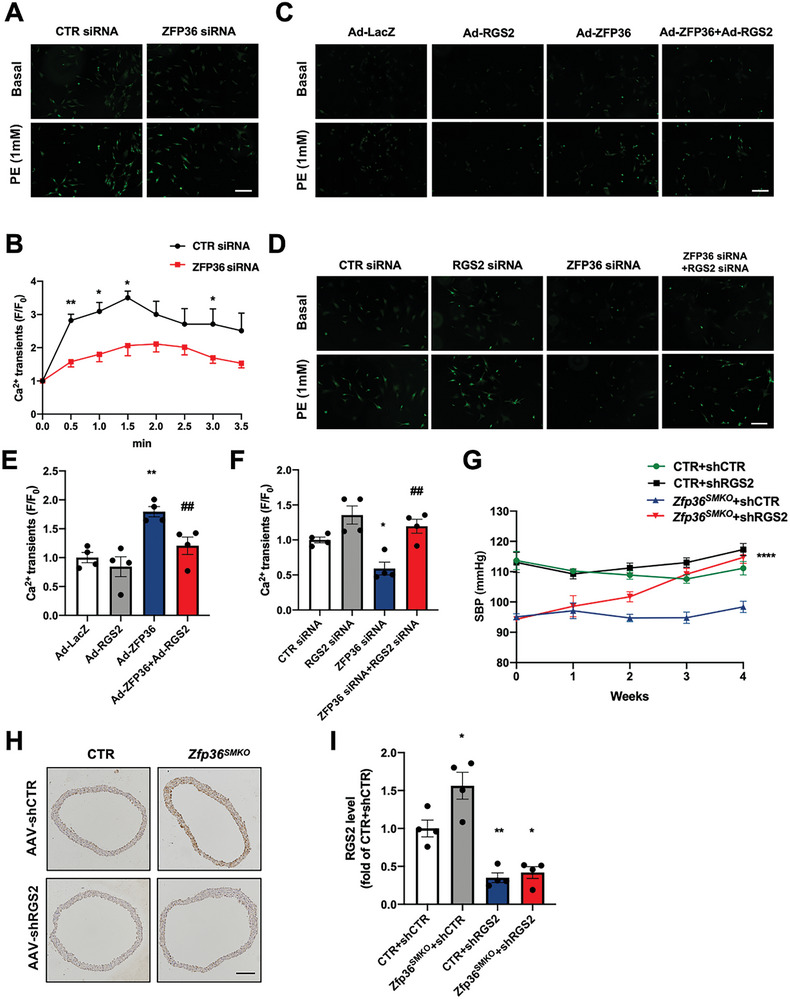
ZFP36 regulated GPCR‐mediated intracellular Ca^2+^ through RGS2. A) VSMCs were transfected with control siRNA or ZFP36 siRNA, loaded with the green fluorescent calcium indicator Fluo‐4 AM, and treated with 1 mmol L^−1^ phenylephrine (PE) to measure cytosolic Ca^2+^ levels by fluorimetry. B) Fluorescent changes were quantified at different times of (A) ^*^ *P* < 0.05, ^**^ *P* < 0.01 *vs* CTR siRNA. C) VSMCs were infected with adenovirus‐expressing GFP, ZFP36, and/or RGS2, followed by cytosolic Ca^2+^ measurement by fluorimetry. D) VSMCs were transfected with CTR, ZFP36, and/or RGS2 siRNA, followed by cytosolic Ca^2+^ measurement by fluorimetry. E,F) Quantitative Ca^2+^ fluorescence after phenylephrine treatment of (C,D). Data were expressed as the ratio of the maximum to minimum fluorescence value by normalization to fluorescent intensity in control cells (*n =* 4). ^**^ *P* < 0.01 *vs* Ad‐LacZ, ^##^ *P* < 0.01 *vs* Ad‐ZFP36 (E). ^*^ *P* < 0.05 *vs* CTR siRNA, ^##^ *P* < 0.05 *vs* ZFP36 siRNA (F). G) CTR, and *Zfp36^SMKO^* mice received tail‐vein injections of AAV9‐shRNA targeting control or RGS2 shRNA, then blood pressure was monitored (*n =* 5), ^****^ *P* < 0.0001 *vs Zfp36^SMKO^ +* sh CTR. H,I) Immunohistochemical staining and quantitative analysis of RGS2 protein in aortas (*n =* 4). Scale bar: 100 µm. ^*^ *P* < 0.05, ^**^ *P* < 0.01 *vs* CTR + shCTR. Data were expressed as the mean ± SEM. Two‐tailed Student's unpaired *t*‐test was used for B, one‐way ANOVA followed by Tukey's post‐test analysis was used for (E, F, I), and repeated measures ANOVA was used for G.

Given that Ca^2+^ is the key molecule in excitation‐contraction signaling pathways, we explored whether ZFP36 affects VSMC contraction by regulating intracellular Ca^2+^ concentration. VSMCs were transfected with an siRNA targeting ZFP36 and subsequently treated with 1 mm phenylephrine (PE), followed by Ca^2+^ measurement (**Figure** **6**A). PE treatment led to a transient increase in cytosolic calcium levels, which was inhibited by ZFP36 knockdown (Figure 6B). RGS2 is a negative regulator of GPCRs. To determine whether ZFP36 regulates the GPCR‐mediated intracellular Ca^2+^ increase via RGS2, VSMCs were infected with adenoviruses expressing ZFP36 and/or RGS2. ZFP36 overexpression increased the fluorescence intensity of Ca^2+^ induced by PE, which was alleviated by RGS2 overexpression (Figure 6C,E). Similarly, RGS2 knockdown reversed the decreased fluorescence intensity of Ca^2+^ in ZFP36‐deficient VSMCs (Figure 6D,F), suggesting that ZFP36 regulates the GPCR‐mediated increase in intracellular Ca^2+^ through RGS2. The in vivo injection of AAV9‐shRNA targeting RGS2 restored the decreased blood pressure in *Zfp36^SMKO^* mice (Figure 6G). Moreover, immunohistochemical staining revealed a decrease in RGS2 protein levels in the aortic smooth muscle after injection of the RGS2 shRNA virus (Figure 6H,I). Collectively, these results indicate that RGS2 plays an important role in the ZFP36‐mediated regulation of VSMC contraction and blood pressure.

### 2.7 VSMC‐Specific ZFP36 Knockout Attenuates AngII‐Induced Hypertension and Vascular Remodeling

**Figure 7:**
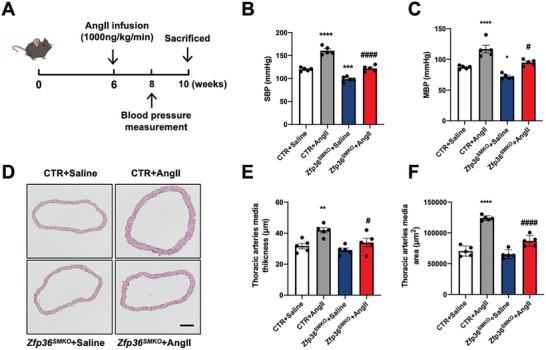
VSMC‐specific ZFP36 knockout significantly attenuated AngII‐induced hypertension and vascular remodeling. A, CTR, and *Zfp36^SMKO^* mice were chronically treated with AngII (1,000 ng kg^−1^ min^−1^) at the age of 6 weeks. B,C), CTR, and *Zfp36^SMKO^* mice were infused with AngII for two weeks and blood pressure was measured by the tail‐cuff method (*n =* 5). D) After infusing with AngII for four weeks, representative hematoxylin‐eosin staining images of thoracic arteries from CTR and *Zfp36^SMKO^* mice with or without AngII treatment. Scale bar: 100 µm. E,F) Statistical analysis of media wall thickness (E, *n* = 5) and area of aortas (F, *n =* 5). ^**^ *P* < 0.01, ^****^ *P* < 0.0001 *vs* CTR + Saline, ^#^ *P* < 0.05, ^####^ *P* < 0.0001 *vs* CTR + AngII. Data were expressed as the mean ± SEM. One‐way ANOVA followed by Tukey's post‐test analysis was used for (B, C, E, and F).

To explore whether ZFP36 is involved in the pathogenesis of hypertension, control and *Zfp36^SMKO^* mice were chronically treated with AngII (1,000 ng kg^−1^ min^−1^) (**Figure** **7**A). After two weeks of treatment, *Zfp36^SMKO^* mice exhibited lower systolic blood pressure and mean blood pressure levels than control mice (Figure 7B,C). After four weeks, AngII‐induced arterial remodeling, characterised by an increased media layer thickness and area of thoracic arteries, was notably alleviated in *Zfp36^SMKO^* mice compared to control mice (Figure 7D–F). Thus, VSMC ZFP36 plays an important role in the pathogenesis of hypertension.

### 2.8 AAV9‐Mediated ZFP36 Knockdown Ameliorates Hypertension in SHR Rats

**Figure 8:**
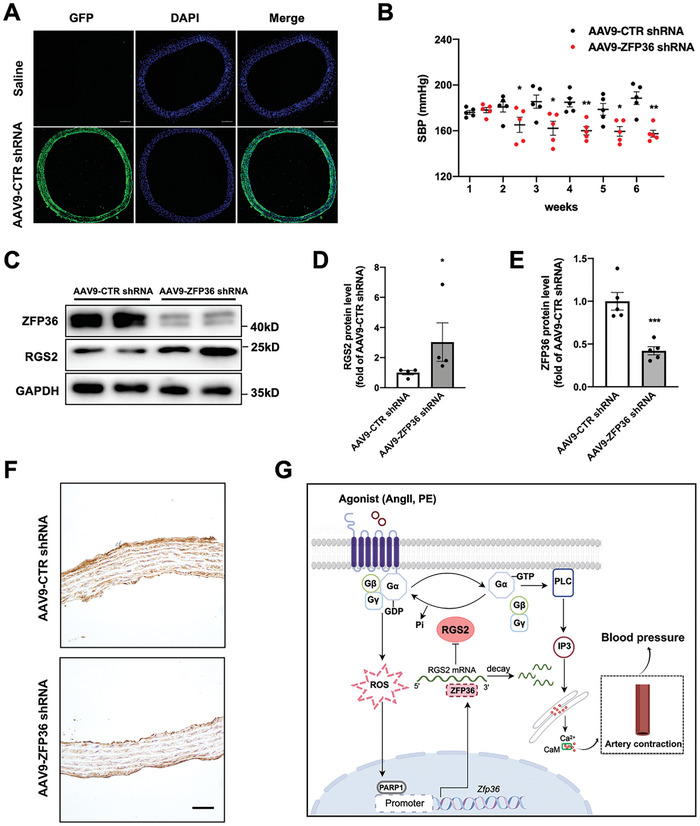
AAV9‐mediated ZFP36 knockdown ameliorated hypertension in SHR rats. A–F) SHR rats were injected with AAV9‐shRNA targeting control (CTR) or ZFP36, immunofluorescent staining of GFP in thoracic arteries from injected with saline and AAV9‐CTR shRNA with GFP (A). Scale bar: 100 µm. Systolic blood pressure was measured by the tail‐cuff method in rats (B, *n =* 5). Western blot analysis of RGS2 (D, *n* = 4) and ZFP36 (E, *n* = 5) levels in rat aortas (C). Immunohistochemical staining of ZFP36 protein in rat aortas (F). ^*^ *P* < 0.05, ^**^ *P* < 0.01, ^***^ *P* < 0,001 *vs* AAV‐CTR shRNA. Data were expressed as the mean ± SEM. Two‐tailed Student's unpaired *t*‐test was used for (B, D, and E). G) Proposed model for the role of ZFP36 in the pathogenesis of hypertension. In VSMCs, AngII activated poly (ADP‐ribose) polymerases1 (PARP1) to induce *Zfp36* expression at the transcription level. ZFP36 reduced RGS2 expression by impairing its mRNA stability, and promoted GPCR‐mediated intracellular Ca^2+^ increase and subsequent smooth muscle contraction.

To evaluate whether ZFP36 deficiency in VSMCs affects the blood pressure of SHR rats, we prepared and injected AAV9 expressing rat control (CTR) or ZFP36 shRNA under the control of the SM22 promoter into SHR rats. After AAV9‐CTR shRNA with green fluorescence protein (GFP) infection, we detected the delivery capability of AAV9 to media layer of arteries, which showed that smooth muscle cells were strongly positive for GFP, suggesting that AAV9 could be delivered in aorta (**Figure** **8**A). The injection of AAV9‐shRNA targeting ZFP36 ameliorated hypertension in SHR rats (Figure 8B). ZFP36 protein levels in the smooth muscle were significantly decreased in the aortas of rats injected with AAV9‐ZFP36 shRNA compared to those injected with AAV9‐CTR shRNA, while the RGS2 protein levels were upregulated (Figure 8C–E). Furthermore, immunohistochemical staining aslo confirmed that ZFP36 level was decreased in the media layer of aorta in rats which injected with AAV9‐ZFP36 shRNA (Figure 8F). Thus, targeting ZFP36 is a potential strategy for the treatment of hypertension.

## 3 Discussion

Hypertension is a leading contributing factor to all‐cause global mortality and predisposes individuals to heart failure, renal failure, peripheral vascular disease, and other medical conditions. Modern medicine can effectively monitor and maintain normal blood pressure in most individuals; however, some patients are resistant to current therapies. Therefore, a deeper understanding of the exact mechanisms responsible for hypertension is crucial. In this study, we established a functional and mechanistic link between smooth‐muscle ZFP36 and blood pressure. Specifically, ZFP36 levels were increased in the arteries of patients and animals with hypertension. According to in vitro experiments, AngII enhanced the transcription of *Zfp36* via PARP1. By generating conditional knockout mice, we demonstrated that ZFP36 deletion in VSMCs reduced blood pressure levels and arterial contractility. Mechanistically, ZFP36 reduced RGS2 expression by impairing its mRNA stability and promoting GPCR‐mediated intracellular Ca^2+^ increase and subsequent smooth‐muscle contraction (Figure 8G). Moreover, the VSMC‐specific depletion of ZFP36 inhibited AngII‐induced hypertension and vascular remodeling in mice, whereas ZFP36 deficiency attenuated hypertension in SHR rats. Overall, our findings suggest that ZFP36 plays a crucial role in smooth muscle contraction and maintaining blood pressure.

The role of ZFP36 as a key factor in post‐transcriptional gene regulation has previously been established, especially regarding its function in promoting the mRNA decay of ARE‐containing genes, many of which are implicated in immune responses and tumorigenesis. ZFP36‐knockout mice develop severe inflammatory syndrome with destructive arthritis, cachexia, myeloid hyperplasia, and autoimmunity.^[^ ^17^ ^]^ Moreover, ZFP36 activity was tightly regulated during inflammation. Although ZFP36 is inactivated during the onset of inflammation, it is gradually reactivated during the progression of inflammation via autoregulatory feedback loops.^[^ ^18^, ^19^ ^]^ Cxcl1 and Cxcl2 show a strong increase in the expression in ZFP36‐deficient bone marrow‐derived macrophages during peak inflammation.^[^ ^20^ ^]^ In addition, other chemokines including Ccl2,^[^ ^21^ ^]^ Ccl3,^[^ ^22^ ^]^ and Ccl4^[^ ^23^ ^]^ are downregulated by ZFP36 at the start of the resolution phase. In addition to its role in inflammation, numerous studies over the last decade have reported the role of ZFP36 in carcinogenesis. For example, ZFP36 has tumour‐suppressive properties that are directly related to its ability to post‐transcriptionally regulate oncogenic mRNAs, including NOTCH1, MYC, BCL‐2, and COX‐2.^[^ ^24^, ^25^, ^26^ ^]^ ZFP36 can also complement the function of tumour suppressors such as p53 and LATS2.^[^ ^27^, ^28^ ^]^ In this study, we found that *Zfp36^SMKO^* mice developed a phenotype with decreased blood pressure under both physiological and pathological conditions by regulating the contraction of VSMCs, which enhances our understanding of the functions of ZFP36.

AngII is the downstream active peptide of the renin–angiotensin system that promotes VSMC contractility, proliferation/migration, and calcification by binding to its receptors.^[^ ^29^, ^30^ ^]^ AngII levels are elevated in the circulation of patients with diabetes and are important risk factors for hypertension and vascular diseases.^[^ ^31^, ^32^ ^]^ AngII can increase cellular calcium in VSMCs via the influx of extracellular calcium and the release of internal calcium, which stimulates the contractility of the vessel.^[^ ^33^ ^]^ The GPCR signaling pathway also plays an important role in the AngII‐induced elevation of cellular calcium in VSMCs. In this study, we found that AngII induces ZFP36 expression, which elevates GPCR‐mediated intracellular Ca^2+^ levels via RGS2. Therefore, our study reveals a novel mechanism of AngII‐associated hypertension and vascular diseases.

As a major component of the tunica media of blood vessels, VSMC contraction plays an important role in the regulation of peripheral vascular resistance and blood pressure. Vascular dysfunction, excessive vasoconstriction, and vasospasm can lead to major cardiovascular disorders such as hypertension and coronary artery disease. Over the past few decades, important studies and major discoveries have helped better understand the relationship between VSMC contractility and blood pressure. Hippo pathway effectors yes‐associated protein 1 and WW domain‐containing transcription regulator 1 impair agonist‐stimulated vascular contractility and myogenic responsiveness by regulating genes involved in VSMC differentiation and contractile function.^[^ ^34^ ^]^ Orphan G Protein–Coupled Receptor GPRC5B controls smooth muscle contractility and differentiation by inhibiting prostacyclin receptor signaling.^[^ ^35^ ^]^ Furthermore, VSMC‐specific deletion of the family with sequence similarity 3 in mice reduces vessel contractility and blood pressure levels by modulating the ATP signaling pathway.^[^ ^36^ ^]^ Besides to ZFP36, our previous study found that HuR, another RNA binding protein involved in regulating mRNA stability by binding adenylate‐uridylate–rich elements, could regulate the contraction of vascular smooth muscle and maintain blood pressure.^[^ ^10^ ^]^ Overall, identifying the mechanisms of VSMC contraction is a major research goal for determining the causes of vascular dysfunction and exaggerated vasoconstriction in vascular diseases.

In summary, our study revealed that ZFP36 plays a crucial role in the regulation of vascular constriction and blood pressure by modulating the GPCR signaling pathway to maintain calcium homeostasis in VSMCs. Overactivation of the PARP1‐ZFP36‐RGS2 pathway in VSMCs is an important novel mechanism in AngII‐related hypertension and vascular diseases. Moreover, ZFP36 knockout alleviated AngII‐induced hypertension and vascular remodeling in mice, whereas decreased ZFP36 expression ameliorated hypertension in spontaneously hypertensive rats. Given the key role of ZFP36 in blood pressure, ZFP36 inhibitors have potential applications as a therapeutic strategy to treat hypertension and vascular diseases in humans.

## 4 Experimental Section

### Animals

*Zfp36*‐floxed (*Zfp36^fl/fl^*) mice were obtained from Professor Pavel Kovarik at the University of Vienna.^[^ ^37^ ^]^ Mice expressing Cre recombinase under the control of the SM22 promoter (*SM22‐Cre*) were obtained from Cyagen Biosciences, Inc. Spontaneously hypertensive rats (SHRs) and Wistar rats were purchased from Beijing Vital River Laboratory Animal Technology Co. VSMC‐specific ZFP36‐knockout (*Zfp36^flox/flox^*/*Cre^+^*; *Zfp36^SMKO^*) mice were generated by crossbreeding *Zfp36^flox/flox^* mice with SM22‐Cre transgenic mice. Littermate *Zfp36^flox/flox^*/*Cre^−^* mice were used as controls. For the hypertension model, male mice were subcutaneously implanted with mini‐pumps (#Alzet model 2004; Alzet Scientific Products) that released AngII (1,000 ng kg^−1^ min^−1^, #HY‐13948; MCE) or saline for 28 days. Animals were housed under standard laboratory conditions with the temperature maintained at 24 °C and a 12‐h light‐dark cycle. For anesthetizing mice, ketamine (100 mg kg^−1^ body weight) and xylazine (10 mg kg^−1^) were intraperitoneally injected. For sacrificing mice, CO_2_ asphyxiation was used. All experiments were conducted in accordance with the protocols approved by the Animal Care and Use Committee of Shandong University (No. KYLL‐2022(ZM)‐008).

### Isolation of Primary VSMCs

Mice were immediately perfused with phosphate‐buffered saline (PBS) after sacrifice. The aorta was aseptically isolated and removed the adventitia and scraped the inner wall. Tissue was washed three times in PBS, placed into an enzymatic dissociation cocktail 2 U ml^−1^ Liberase (#5401127001; Sigma) and 2 U ml^−1^ elastase (#LS002279; Worthington) in Hank's Balanced Salt Solution (HBSS) and minced. After incubation at 37°C for 1h, the cell suspension was strained and then pelleted by centrifugation at 500 g for 5 min. After centrifugation, cells were reseeded in a T25 tissue culture flask. Isolated VSMCs were cultured in high glucose Dulbecco Modified Eagle Medium (DMEM) containing 10% fetal bovine serum (FBS), 100 U ml^−1^ penicillin, and 100 µg ml^−1^ streptomycin, and cells at passages 3 to 6 were used for further experiments.

### Single‐Cell Sequencing Dataset Analysis

The dataset GSE149777 was re‐analyzed using the method described in the original literature. R package Seurat (version4.4.0) was used for cell filtration, data normalization, dataset integration, dimension reduction, and cell clustering and cluster visualization with default parameters unless otherwise specified. For sub‐clustering, gene count of cells from the same major cell type were retrieved and integrated using 20 D. Cell type‐specific and subcluster‐specific markers were found with FindAllMarkers function in Seurat with arguments avg_log_2_FC > 0.25, min.pct > 0.1 and only.pos = TRUE. adj.*p*‐value < 0.05 determined by Wilcoxon rank‐sum tests was used to further filter for significantly enriched genes. Differentially expressed genes between indicated cell types or sub‐clusters were found with the FindMarkers function in Seurat with similar conditions as the FindAllMarkers function.

### Immunofluorescent Staining

Fresh vascular tissues were harvested, embedded in OCT compound, and cryosectioned. Non‐specific binding sites were blocked in 5% goat serum for 1 h at room temperature. Sections were then incubated with primary antibodies of rabbit anti‐α smooth muscle actin (SMA) antibody (1:100, #Ab5694, Abcam) or mouse anti‐ZFP36 antibody (1:100, #AB‐2725702, Invitrogen) overnight at 4 °C, then with the secondary antibody of FITC‐conjugated goat anti‐rabbit IgG (#SA00006‐2, Proteintech) and CY3‐conjugated goat anti‐mouse IgG (#SA00006‐3, Proteintech) at 37 °C for 2 h. Finally, nuclei were stained with DAPI (4', 6‐diamidino‐2‐phenylindole) for 5 min at room temperature, and immunofluorescence was analyzed under a fluorescent microscope.

### Hematoxylin/Eosin (HE) Staining and Immunohistochemistry

Tissues were fixed in 4% paraformaldehyde for 48 h, embedded in paraffin, and cut into transverse sections 5‐µm thick. The sSections were dewaxed with xylene, rehydrated with ethanol in descending order, rinsed with distilled water, and stained with HE eosin. For immunohistochemistry, after antigen repair and peroxidase removal from vascular tissues, the sections were incubated with primary antibodies at 4 °C overnight. After washing three times with PBS, the sections were treated with horseradish peroxidase‐conjugated secondary antibodies. Subsequently, 3‐amino‐9‐ethylcarbazole was added to the mixture. Images were taken by Nikon microscopy.

### Wire Myography

Wire myography experiments were performed using a four‐channel myograph system (DMT630MA, Danish Myo Technology). Secondary branches of the mesenteric arteries from control and *Zfp36^SMKO^* mice were cleaned of connective tissue and cut into segments. Isolated arteries were suspended in the wire myograph, and set to a preload tension of 2.5–3.0 mN then equilibrated for 30 min in Krebs solution (120.0 mmol L^−1^ NaCl, 5.0 mmol L^−1^ KCl, 25.0 mmol L^−1^ NaHCO_3_, 11.0 mmol L^−1^ glucose, 1.0 mmol L^−1^ KH_2_PO_4_, 1.2 mmol L^−1^ MgSO_4_, and 1.0 mmol L^−1^ MgCl_2_, pH 7.4) at 37°C. After stimulation with 124.0 mmol L^−1^ KCl three times, arteries were treated with norepinephrine (NE, 10 µmol L^−1^), endothelin‐1 (ET‐1, 0.1 µmol L^−1^), angiotensin II (AngII, 10 µmol L^−1^), and U46619 (0.1 µmol L^−1^).

### Blood Pressure Measurements

Blood pressure was measured using radiotelemetry and the tail‐cuff method. For radiotelemetry, the mice were anesthetized with 1–2% isoflurane. The left carotid artery was isolated and a catheter (PE10 tubing) was inserted. The body of the telemetry transmitter unit (TA11PA‐C10; Data Sciences International, St. Paul, MN, USA) was placed subcutaneously on the left side of the abdomen, and the signal was received and recorded using a data acquisition program (Dataquest ART 3.1; Data Sciences International). For the tail‐cuff method, animals were kept in restraint tubes and their tails were passed through the sensor. The hop pockets were kept in a prewarmed box at 37 °C, and blood pressure was measured for 20 min at the same time every day. Animals were acclimated to the system for seven consecutive days prior to blood pressure measurement (#BP‐2010E, Softron).

### RNA Immunoprecipitation (RIP) Assay

The RNA immunoprecipitation (RIP) assay was performed using a Magna RIP kit (#17‐700, Millipore). Briefly, cell lysates were incubated overnight at 4 °C with protein A/G beads pre‐conjugated with 5 µg rabbit IgG or ZFP36 antibody (#AB2725702, Invitrogen). RNA was then isolated from the immunoprecipitates followed by PCR using *Rgs2* primers: 5′‐ATCAAGCCTTCTCCTGAGGAA‐3′ and 5′‐GCCAGCAGTTCATCAAATGC‐3′.

### Chromatin Immunoprecipitation (ChIP) Assay

Chromatin immunoprecipitation (ChIP) assays were performed with SimpleChIP® Enzymatic Chromatin IP Kit (Cell Signaling technology) according to the manufacture's protocol. VSMCs were cross‐linked using 1% formaldehyde, fragmented by sonication, and immunoprecipitated. For each immunoprecipitate, 5 µg of anti‐PARP1 antibody (#ab227244, Abcam) and 5 µg of total rabbit IgG was incubated with the diluted sheared chromatin. The final ChIP DNA was then used as the template for reversetranscription quantitative PCR (RT‐PCR) reactions, using primers targeting the *Zfp36* promoter as follows: ‐1261:5′‐TTCAGGTTTAAGTTTTTATTTCGCC‐3′ and 5′‐TAGAGCCAACCCTATTATAGAAAGA‐3′; ‐436:5′‐CCAGCAGATCCTACTTGTGAATGTC‐3′ and 5′‐CAAGTTGGAAGAGACAGAGATGGCC‐3′.

### Luciferase Reporter Assays

Luciferase reporter transfection and dual‐luciferase assays were performed using a Dual‐Luciferase Reporter Gene Assay Kit (#RG027; Beyotime). Briefly, VSMCs were seeded in 24‐well plates and transfected using Lipofectamine 3000 (#L3000075, Invitrogen) with 100 ng of reporter vector containing the indicated target sequences; an empty vector was used as the control. The PRL‐TK vector was co‐transfected as an internal control. After 48 h of transfection, the cells were lysed using a passive lysis buffer and subjected to the luciferase assay according to the manufacturer's protocol.

### Calcium Measurement

After being washed with PBS three times, VSMCs were loaded with 4 µmol L^−1^ of Fura‐4AM (as a molecular probe) in Ca^2+^‐free Hank's Balanced Salt Solution containing 0.02% Pluronic F‐127 (KeyGEN BioTECH) then cultured in an incubator for 30 min. Finally, the cells were exposed to 1 mmol L^−1^ phenylephrine. Fluorescent images of individual cells were acquired using fluorescence microscopy at 1 frame/10 s for at least 300 s after phenylephrine stimulation. The changes in calcium ion levels were calculated as the ratio of the average fluorescence intensity of a single cell to its initial value (before phenylephrine addition).

### Antibodies and Reagents

Antibodies targeting ZFP36 (AB2725702, Invitrogen, Immunofluorescent staining), ZFP36 (71632S, Cell Signaling Technology, Western blot), PARP1 (ab227244, Abcam), RGS2 (Abs136141, Absin Bioscience Inc), α‐SMA (ab5694, Abcam), and GAPDH (5174S, Cell Signaling Technology) were obtained commercially. Adenovirus‐overexpressing ZFP36, RGS2, and green fluorescent protein (GFP) control vectors were purchased from Vigene Bioscience. Adeno‐associated virus 9 (AAV9) expressing rat ZFP36 and RGS2 shRNAs under the control of the SM22 promoter were purchased from Vigene BioSciences. siRNAs targeting PARP1, ZFP36, and RGS2 were purchased from RibiBio (Guangzhou, China).

### Quantitative Real‐Time PCR (RT‐PCR)

RNA was isolated from VSMCs or aortas using the TRIzol reagent (#15596018, Invitrogen). Further, 0.5‐1.0 µg of total RNA was used for the reverse transcription reaction. RT‐PCR was performed using TB Green Premix Ex Taq II (#RR820A; TaKaRa Bio). The primers were as follows: ZFP36:5′‐GTCACCCTCACCTACTTCGC‐3′ and 5′‐ACTTGTGGCAGAGTTCCGTT‐3′; RGS2:5′‐ATCAAGCCTTCTCCTGAGGAA‐3′ and 5′‐GCCAGCAGTTCATCAAATGC‐3′; GAPDH: 5′‐TTGTCAAGCTCATTTCCTGGTATG‐3′ and 5′‐GCCATGTAGGCCATGAGGTC‐3′.

### Western Blot Analysis

Total proteins were extracted from the aortas or VSMCs in RIPA lysis buffer, and protein concentrations were measured using a BCA Protein Assay Kit (#PC0020; Solarbio). Proteins were separated using sodium dodecyl sulphate‐polyacrylamide gel electrophoresis and transferred to nitrocellulose membranes. After blocking with 5% non‐fat milk, membranes were incubated with primary antibodies (anti‐ZFP36: 0.5 µg ml^−1^; anti‐RGS2: 1 µg ml^−1^) at 4 °C overnight then with horseradish peroxidase‐labelled secondary antibody for 1 h at room temperature, followed by detection using enhanced chemiluminescence (Amersham Imager 680; GE).

### Human Cases

Aortic samples were collected from the autopsies of male patients with normotension and hypertension. Mesenteric artery samples from male patients with normotension and hypertension were collected during subtotal gastrectomy. All procedures involving human subjects were performed following the Declaration of Helsinki and were approved by the Medical Institutional Ethics Committee of Qilu Hospital, Shandong University (No. KYLL‐2022(ZM)‐009). Informed consent was obtained from each participant.

### Statistical Analysis

Data were expressed as the mean ± SEM and analyzed using GraphPad Prism 8.0 and SPSS 23.0 (IBM Software). The Shapiro–Wilk test was performed to test the normality of the data. Statistical differences were evaluated using the Student's *t*‐test for data classified into two groups, and one‐way analysis of variance (ANOVA) for data classified into three or more groups (Tukey's post‐test). *P* < 0.05 was considered statistically significant.

## Conflict of Interest

The authors declare no conflict of interest.

## Author Contributions

X.C. and Y.W. contribute equally to this work. W.Z. designed the research; X.C., Y.W., H.L., and L.W. performed the research; X.C., Y.W., X.X., and S.Z. analyzed the data; P.K. supported the animals; W.Z., X.C., H.L., L.W., and S.L. wrote the manuscript; W.Z., S.L., S.L., Q.Z., J.Y., C.Z., J.T., and Y.L. critically reviewed the manuscript and provided important intellectual content. All authors reviewed and commented on this manuscript.

## Supporting information



Supporting Information

## Data Availability

The data that support the findings of this study are available on request from the corresponding author. The data are not publicly available due to privacy or ethical restrictions.
