# Supplementary material for: ZFP36 Regulates Vascular Smooth Muscle Contraction and Maintains Blood Pressure
Source: Adv Sci (Weinh). 2024 Nov 26;12(3):2408811. doi: 10.1002/advs.202408811 (PMC11744710; doi:10.1002/advs.202408811)
Supplement: Supplementary file 1 — Supporting Information [file ADVS-12-2408811-s001.docx]

Supporting Information

**ZFP36 Regulates Vascular Smooth Muscle Contraction and Maintains Blood Pressure**

*Xiuru Cui^#^, Yawei Wang^#^, Hanlin Lu, Lei Wang, Xianwei Xie, Shenghao Zhang, Pavel Kovarik, Shuijie Li, Qunye Zhang, Jianmin Yang, Cheng Zhang, Jinwei Tian*, Yan Liu*, Wencheng Zhang**

**
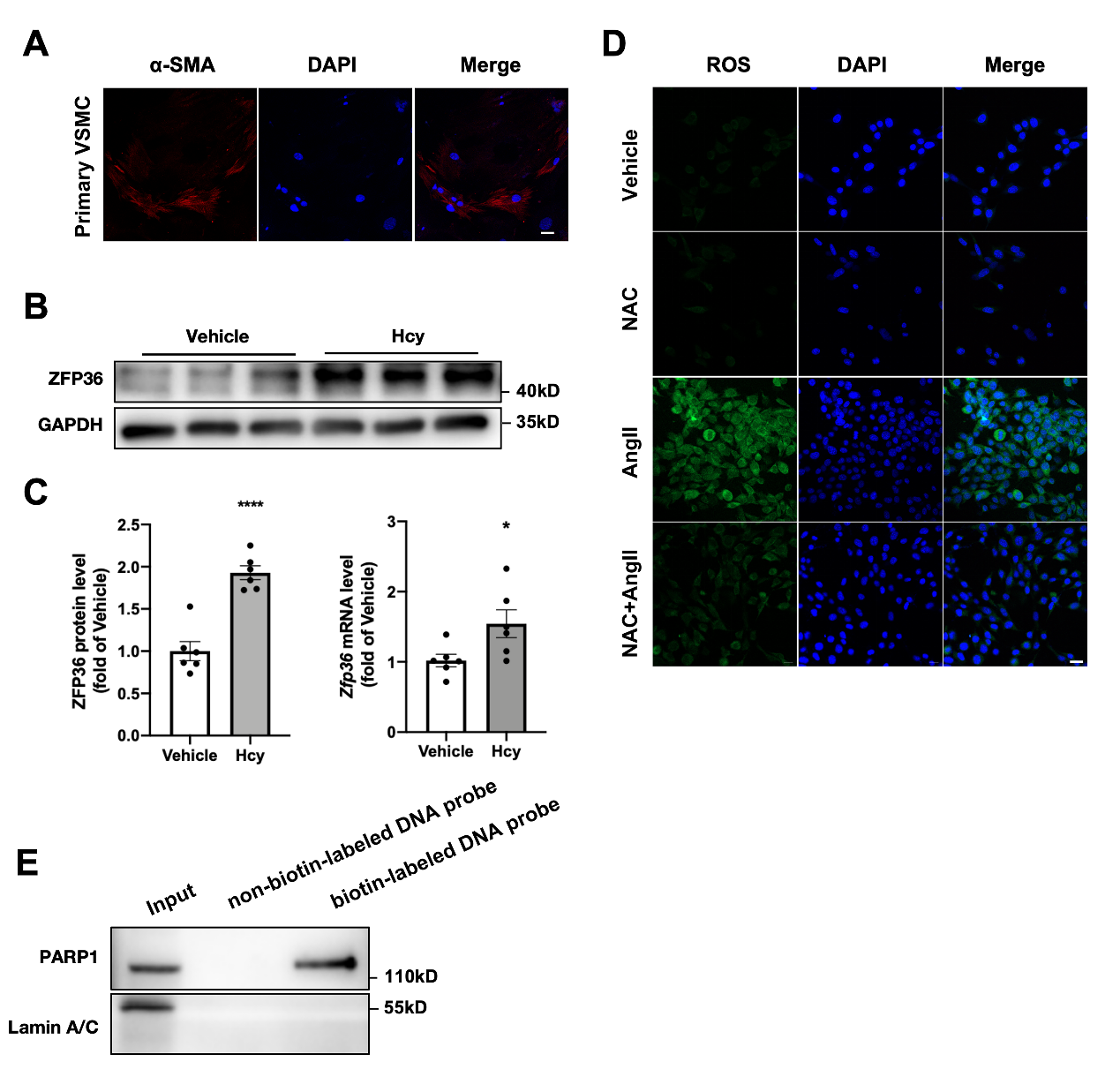
**

Figure S1. AngII and Hcy upregulates ZFP36 expression in VSMCs. A) Immunofluorescent staining of α-SMA in cultured mouse primary vascular smooth muscle cells (VSMCs). Scale bar: 25 μm. B-C) VSMCs were treated with Homocysteine (Hcy, 1 μmol L^-1^) for 48h followed by western blot analysis (*n* = 6) and RT-PCR analysis (*n* = 5) of ZFP36 expression. **P* < 0.05, **** *P* <0.0001 vs Vehicle. D) VSMCs were pretreated with N-acetylcysteine (NAC, 1 mmol L^-1^) for 30 min and then stimulated with AngII followed by ROS staining. Scale bar: 25 μm. E) DNA pull down with a biotin-labeled DNA probe specific to the Zfp36 promoter region and non-biotin labeled DNA probes as controls was performed followed by western blot analysis. Data were expressed as the mean ± SEM. Two-tailed Student’s unpaired t-test was used for C.


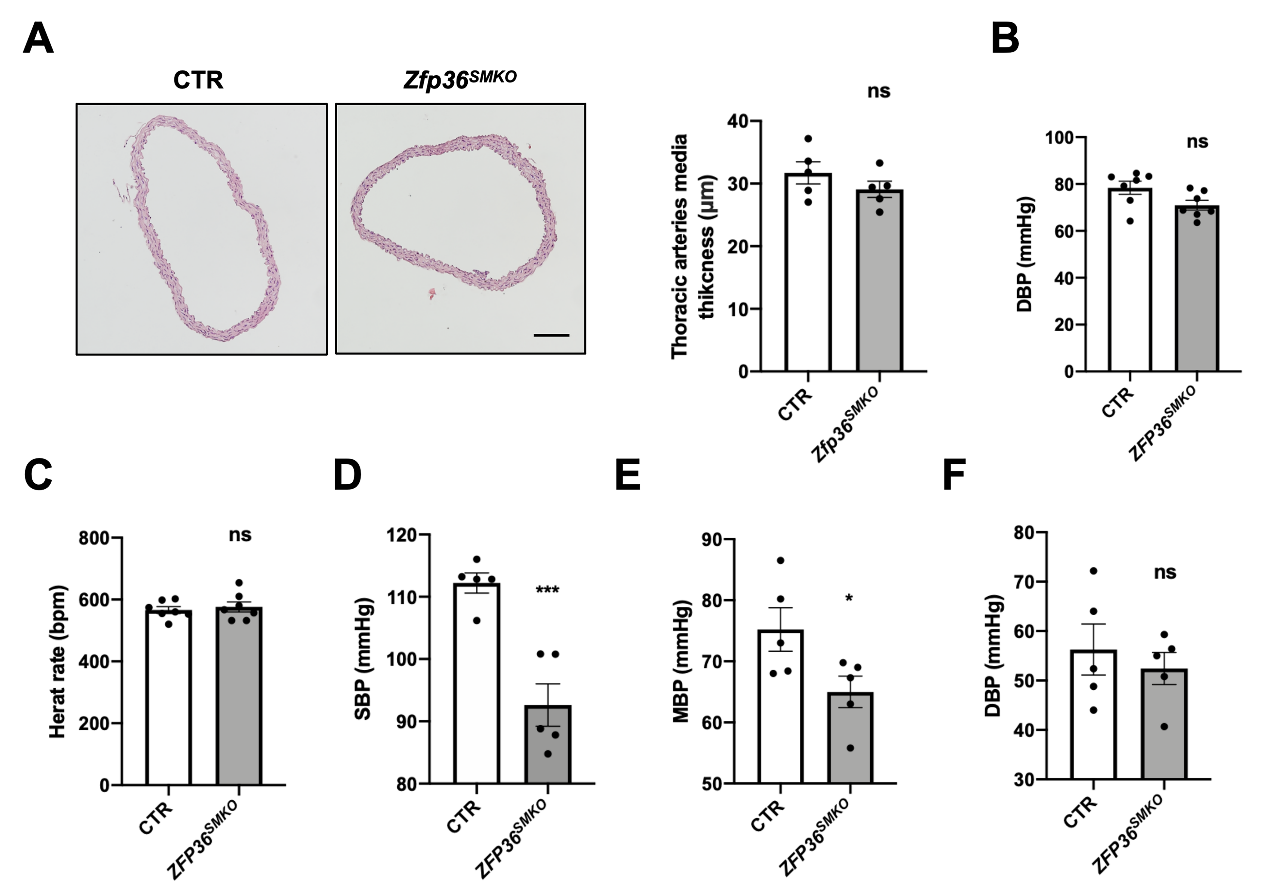


Figure S2. VSMC-specific knockout of ZFP36 reduces blood pressure in mice. A) Representative hematoxylin-eosin staining images of thoracic arteries and statistical analysis of media wall thickness from CTR and *Zfp36^SMKO^* mice (*n* = 5). Scale bar: 100 μm. B-C) Measurement of the diastolic blood pressure (DBP) (B) and heart rate (C) in male CTR and *Zfp36^SMKO^* mice (*n* = 7). D-F) Measurement of the systolic blood pressure (SBP) (D), mean blood pressure (MBP) (E) and DBP (F) in female CTR and *Zfp36^SMKO^* mice. **P* < 0.05, ****P* < 0.001 *vs* CTR. Data were expressed as the mean ± SEM. Two-tailed Student’s unpaired t-test was used for (A-F).
